# Lanatoside C suppressed colorectal cancer cell growth by inducing mitochondrial dysfunction and increased radiation sensitivity by impairing DNA damage repair

**DOI:** 10.18632/oncotarget.6832

**Published:** 2016-01-07

**Authors:** Mi Ae Kang, Mi-Sook Kim, Wonwoo Kim, Jee-Hyun Um, Young-Joo Shin, Jie-Young Song, Jae-Hoon Jeong

**Affiliations:** ^1^ Research Center for Radiotherapy, Korea Institute of Radiological and Medical Sciences, Seoul, Korea; ^2^ Department of Radiation Oncology, Korea Institute of Radiological and Medical Sciences, Seoul, Korea; ^3^ Korea Mouse Metabolic Phenotyping Center, Lee Gil Ya Cancer and Diabetes Institute, Gachon University, Incheon, Korea; ^4^ Department of Radiation Oncology, Inje University Sanggye Paik Hospital, Seoul, Korea; ^5^ Division of Radiation Cancer Research, Korea Institute of Radiological and Medical Sciences, Seoul, Korea

**Keywords:** lanatoside C, autophagy, mitochondria, DNA damage repair, radiosensitivity

## Abstract

Cardiac glycosides are clinically used for cardiac arrhythmias. In this study, we investigated the mechanism responsible for anti-cancer and radiosensitizing effects of lanatoside C in colorectal cancer cells. Lanatoside C-treated cells showed classic patterns of autophagy, which may have been caused by lanatoside C-induced mitochondrial aggregation or degeneration. This mitochondrial dysfunction was due to disruption of K^+^ homeostasis, possibly through inhibition of Na^+^/K^+^-ATPase activity. In addition, lanatoside C sensitized HCT116 cells (but not HT-29 cells) to radiation *in vitro*. γ-H2AX, a representative marker of DNA damage, were sustained longer after combination of irradiation with lanatoside C, suggesting lanatoside C impaired DNA damage repair processes. Recruitment of 53BP1 to damaged DNA, a critical initiation step for DNA damage repair signaling, was significantly suppressed in lanatoside C-treated HCT116 cells. This may have been due to defects in the RNF8- and RNF168-dependent degradation of KDM4A/JMJD2A that increases 53BP1 recruitment to DNA damage sites. Although lanatoside C alone reduced tumor growth in the mouse xenograft tumor model, combination of lanatoside C and radiation inhibited tumor growth more than single treatments. Thus, lanatoside C could be a potential molecule for anti-cancer drugs and radiosensitizing agents.

## INTRODUCTION

Cardiac glycosides are a large family of naturally derived compounds that contain a steroid nucleus with five- or six-membered lactone ring and sugar moieties. These compounds inhibit the plasma membrane Na^+^/K^+^-ATPase and are clinically used to treat arrhythmia and heart failure. Cardiac glycosides suppress the active counter-transport of Na^+^ and K^+^ across the cell membrane, which leads to an increase in intracellular [Na^+^] and a decrease in intracellular [K^+^] [1]. Recently, many studies have suggested that cardiac glycosides target cancer cells [2–5]. Because human cancer cells tend to express isoforms of the subunits that makeup the Na^+^/K^+^-ATPase, these cells may be sensitive to the cytotoxic effects of cardiac glycosides [5]. Several studies have reported that cardiac glycosides exert several anti-tumor mechanisms, including inhibition of proliferation, induction of apoptosis, and sensitization to chemotherapy, supporting their potential use for cancer therapy [6–8].

Mitochondria supply energy to the cell and are important mediators of apoptosis. Several compounds that destabilize mitochondrial function and cause apoptosis have been identified recently, suggesting mitochondria may be potential targets for anti-cancer drugs. Each type of cancer is complex and caused by different DNA mutations, indicating that cancer treatment with a drug that targets only a few gene products or single pathway is difficult. However, mutations in the mitochondrial genome are very rare, and compounds that target mitochondria may be efficient drugs for treatment of multiple types of cancers [9].

In the present study, we showed that lanatoside C sensitized colorectal cancer cells to radiation *in vitro* and in a mouse xenograft model. Moreover, lanatoside C alone suppressed proliferation of colorectal cancer cells through an apoptosis-independent manner by inducing mitochondrial dysfunction. Activation of these alternative cell death pathways by lanatoside C may be a useful strategy to treat cancer cells that are resistant to apoptosis and may be further exploited for the development of novel cancer therapies.

## RESULTS

### Lanatoside C inhibited the proliferation of colorectal cancer cells

**Figure 1:**
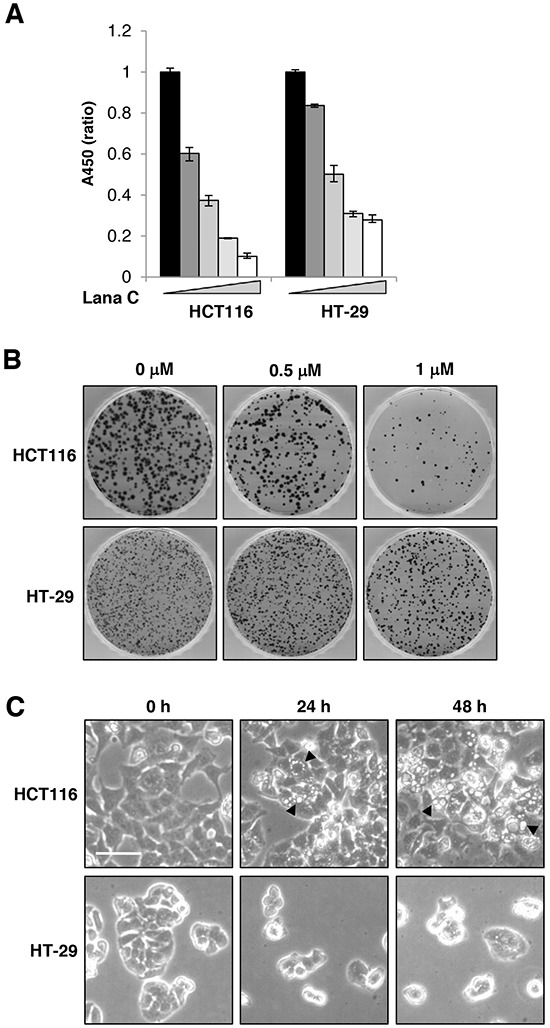
Lanatoside C suppressed proliferation of colorectal cancer cells **A.** HCT116 and HT-29 cells were treated with 0.1, 0.2, 0.5 or 1 μM lanatoside C for 48 h. Cell viability was evaluated using the WST Cell Viability Assay. Data represents the mean ± SD of three independent experiments. **B.** HCT116 and HT-29 cells were treated with lanatoside C for 24 h and surviving colonies were stained with crystal violet after 10 days. **C.** HCT116 and HT-29 cells were treated with 0.5 μM lanatoside C for 24 or 48 h. Morphological changes in the cells were observed. Representative images were obtained at 100× magnification. Scale bar: 50 μm.

Because cardiac glycosides selectively inhibit growth of some malignant cells [10], we first examined the effects of lanatoside C on the proliferation of human colorectal cancer cell lines. Similar to the findings in other types of cancers, lanatoside C significantly inhibited growth of the HCT116 and HT-29 colorectal cancer cell lines (Figure 1A). Because cytotoxicity of conventional anti-cancer drugs is typically due to cell cycle arrest or apoptosis [11], we investigated whether cell cycle arrest or apoptosis contributed to the response to lanatoside C. Supplementary Figure S1 shows that lanatoside C accumulated cells at the G2/M phase of the cell cycle in both cell lines. Cytotoxic effect of lanatoside C was not significant. Propidium iodide positive population was only 10 % and apoptosis markers like cleavage of PARP-1 or caspase 3 was not induced significantly (data not shown). However, as shown in Figure 1B, lanatoside C reduced colony-forming efficiency of both colorectal cancer cell lines, suggesting that lanatoside C caused a replication-dependent cell death instead of physiological cell death. Microscopic observations revealed that lanatoside C induced vacuole formation in HCT116 cells (arrowhead), and the number of vacuoles increased in a time-dependent manner (Figure 1C).

### Lanatoside C induced autophagy in colorectal cancer cells

**Figure 2:**
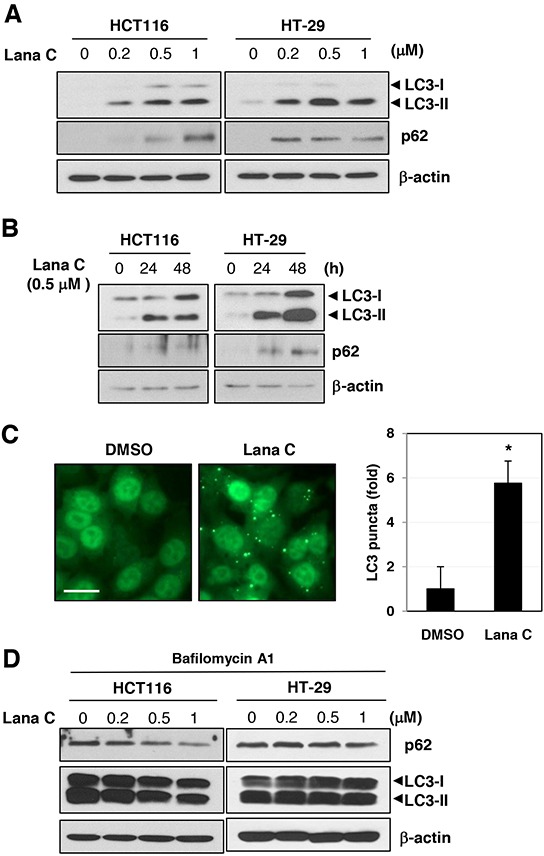
Lanatoside C induced autophagy in colorectal cancer cells **A.** HCT116 and HT-29 cells were treated with the indicated concentration of lanatoside C for 24 h. **B.** HCT116 and HT-29 cells were treated with 0.5 μM lanatoside C for 24 or 48 h. **C.** HCT116 cells expressing GFP-LC3 were treated with 0.5 μM lanatoside and LC3 puncta formation was observed by fluorescence microscopy. Representative images were obtained at 200× magnification. Scale bar: 20 μm. *p < 0.05. **D.** After pretreatment with 0.1 μM bafilomycin A1 for 1 h, HCT116 and HT-29 cells were treated with the indicated concentration of lanatoside C for 24 h. Conversion of LC-I to LC-II and p62 protein level was measured by western blot analysis.

To determine whether vacuole formation was mediated by autophagy, we measured conversion of autophagosome protein LC3-I to LC3-II using western blot analysis. Lanatoside C induced LC3-II form in a dose- and time-dependent manner in both cell lines (Figure 2A and 2B). Another autophagic marker protein, p62/sequestosome 1 (SQTM1), was also induced by lanatoside C treatment. Autophagy induction is often monitored by an assay that depends on translocation of LC3 from the cytosol to newly formed autophagosomes, which appear as cytoplasmic puncta [12]. GFP-LC3-expressing HCT116 cells were incubated in media containing lanatoside C for 48 h. To quantify the autophagy, the number of GFP-LC3 puncta in a cell was measured (Figure 2C). Lanatoside C-treated cells showed significant induction of GFP-LC3 puncta formation. All these results suggested that lanatoside C induced autophagosome formation in colorectal cancer cells. In order to know whether these changes in autophagic flux is due to activation of autophagy or inhibition of autophagosomal degradation, bafilomycin A1 was pre-treated. As LC3-II conversion and p62 accumulation was not further increased by lanatoside C in the presence of bafilomycin A1, lanatoside C impaired autophagosomal degradation (Figure 2D).

### Autophagy induction by lanatoside C was dependent on Erk and JNK MAP kinases

**Figure 3:**
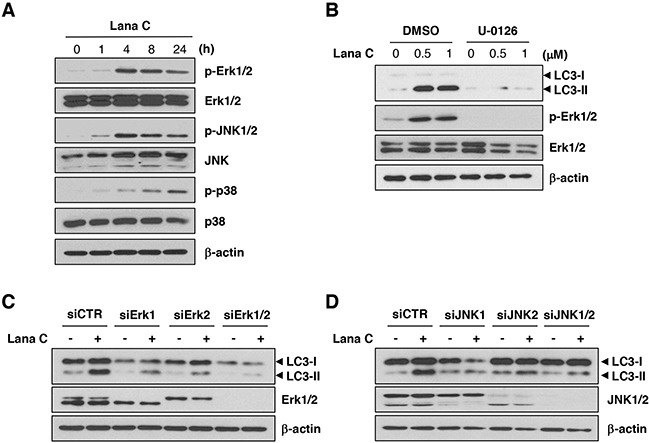
Lanatoside C induced autophagy through Erk1/2 and JNK1/2-mediated mechanisms **A.** HCT116 cells were treated with 1 μM lanatoside C for the indicated times and lysates were prepared. Western blot analysis was performed with antibodies to detect activation of MAPK signaling. **B.** After pretreatment with U-0126 (10 μM) for 1 h, HCT116 cells were treated with the indicated concentration of lanatoside C for 24 h. **C, D.** After transfection with control siRNA (siCTR) or the indicated siRNAs, HCT116 cells were treated with 1 μM lanatoside C for 24 h. Protein expression of LC3, Erk1/2 and JNK1/2 were confirmed by western blot analysis.

Because previous reports show that autophagy is regulated by MAP kinase [13–15], we investigated whether lanatoside C activated MAP kinases. Of three MAPKs, phosphorylation of Erk1/2 and JNK1/2 peaked after 4 h incubation with lanatoside C and decreased thereafter. In contrast, phosphorylation of p38 increased continuously in a time-dependent manner (Figure 3A). To reveal whether MAP kinases were involved in autophagy induction by lanatoside C, we investigated levels of LC3-II after transfection with siRNA against Erk1/2, JNK1/2 or p38. Lanatoside C-induced LC3-II conversion was decreased significantly in the Erk1/2 or JNK1/2-suppressed cells (Figure 3C and 3D). In accordance with this, pretreatment with 10 μM U-0126, an inhibitor of Erk1/2, completely blocked autophagic process induced by lanatoside C (Figure 3B). However, p38 knockdown did not affect LC3-II conversion (data not shown). All these results suggest that lanatoside C induced autophagy via Erk1/2 and JNK1/2 activation.

To investigate whether anti-cancer effect of lanatoside C is mediated by autophagy, essential genes in autophagosome formation were knockdown. Although LC3-II conversion by lanatoside C was blocked by the transfection with ATG5 or Beclin1 siRNA (Supplementary Figure S2A), cell proliferation (Supplementary Figure S2B) and clonogenic cell survival (Supplementary Figure S2C) were not changed with suppression of autophagy. Thus, induction of autophagy does not seem to be a main mechanism of anti-cancer effect of lanatoside C.

### Lanatoside C induced mitochondrial dysfunction in colorectal cancer cells

**Figure 4:**
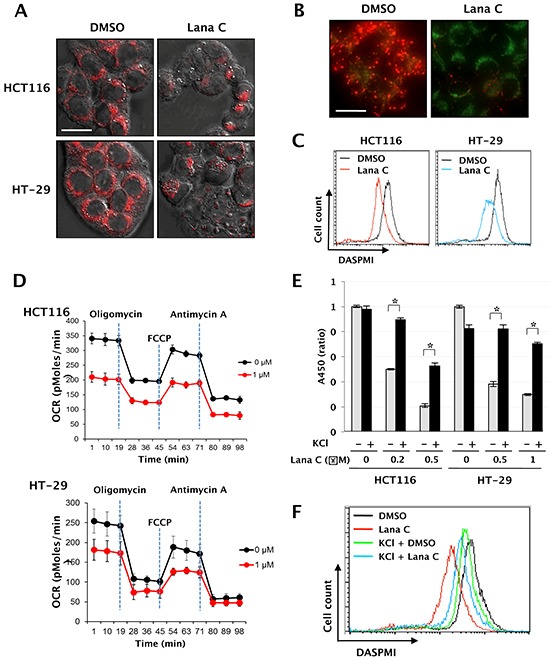
Lanatoside C induced mitochondrial dysfunction by decreasing intracellular [K^+^] level **A.** HCT116 and HT-29 cells were treated with 0 or 1 μM lanatoside C for 24 h. After staining with 20 nM MitoTracker Red CMXROS for 30 min, morphological changes of mitochondria were observed with confocal microscopy. **B.** After pretreatment with lanatoside C (1 μM) for 3 h, HCT116 cells were stained with 10 mg/ml JC-1 for 30 min. Changes in mitochondrial membrane potential were observed with fluorescence microscopy. Representative images were obtained at 200× magnification. Sacle bar: 20 μm. **C.** After pretreatment with lanatoside C (1 μM) for 3 h, HCT116 and HT-29 cells were stained with 10 μM DASPMI for 30 min. Changes in mitochondrial membrane potential were analyzed by flow cytometry. **D.** HCT116 and HT-29 cells were treated with DMSO or 1 μM lanatoside C for 3 h. Oxygen consumption rate (OCR) was measured by using the Seahorse Extracellular Flux Analyzer. Oligomycin, FCCP and antimycin A were injected at the indicated time points. Data are shown as mean ± standard deviation (*n* = 3∼4). **E.** After pretreatment with 25 μM KCl for 1 h, HCT116 and HT-29 cells were treated with lanatoside C for 48 h. Cell viability was quantified with WST Cell Viability Assay. *p < 0.05, control vs. KCl treated group. **F.** After pretreatment with 25 μM KCl for 1 h, HCT116 cells were treated with lanatoside C (1 μM) for 3 h. After staining with 50 μM DASPMI for 30 min, flow cytometry analysis was performed and mitochondrial membrane potential was quantified.

Recent reports show that mitochondria contribute to apoptosis, necrosis, and autophagy, and mitoptosis has been shown to activate autophagy [16]. To investigate effects of lanatoside C on mitochondrial function, we observed morphological changes of mitochondria with MitoTracker staining after treatment with lanatoside C. As shown in Figure 4A, mitochondria were more condensed in lanatoside C-treated cells than in untreated cells. Mitochondrial membrane potential (MtMP) was also examined with JC-1 or DASPMI staining. Polarized mitochondria, as visualized by red JC-1 aggregation puncta, were present in the untreated cells but not the lanatoside C-treated cells (Figure 4B). Coincide with this result, DASPMI staining showed that lanatoside C decreased mitochondrial membrane potential to 49.8 ± 1.4% (Figure 4C).

To verify the effect of lanatoside C on cellular respiration in colorectal cancer cells, we assessed oxygen consumption rate (OCR). To measure maximal oxidative capacity, oligomycin was injected into culture wells prior to injection of the uncoupling agent FCCP. Lanatoside C consistently decreased basal OCR even after 3 h incubation. Lanatoside C decreased oxidative capacity to 68.6 ± 6.7% that of control-treated cells (Figure 4D), suggesting that lanatoside C depolarized the mitochondrial membrane potential and inhibited mitochondrial function.

### Lanatoside C induced mitochondrial dysfunction by disrupting K^+^ homeostasis

Cardiac glycosides exert their effect by inhibiting the Na^+^/K^+^-ATPase, elevating intracellular [Na^+^] and depleting intracellular [K^+^][17]. To determine whether an increase of intracellular [Na^+^] by lanatoside C mediates growth inhibition, we pretreated 25 μM KCl to suppress intracellular Na^+^ accumulation. KCl decreased lanatoside C-mediated cytotoxicity in both cell lines (Figure 4E). Mitochondrial membrane potential in KCl-treated HCT116 cells was approximately 74% of untreated cells. In the presence of lanatoside C, treatment with KCl recovered mitochondrial membrane potential from 41% to 63% of that of untreated cells (Figure 4F).

To determine the signaling between mitochondrial dysfunction and autophagy, mitochondrial membrane potential was checked in MAPKs inhibited cells. Erk1/2 or JNK1/2 knockdown via siRNA transfection or pretreatment with Erk inhibitor (U-0126) or JNK inhibitor (SP600125) did not recover lanatoside C-mediated reduction of mitochondrial membrane potential (Supplementary Figure S3A). Conversely, KCl pretreatment suppressed lanatoside C-induced activation of Erk1/2 and JNK1/2 (data not shown) and GFP-LC3 puncta formation (Supplementary Figure S3B). These data suggested that lanatoside C-induced depolarization of the mitochondrial membrane may be mediated by increased intracellular [Na^+^], followed by activation of Erk1/2 and JNK1/2 and progression of autophagy.

### Lanatoside C increased radiosensitivity by suppressing DNA damage repair

**Figure 5:**
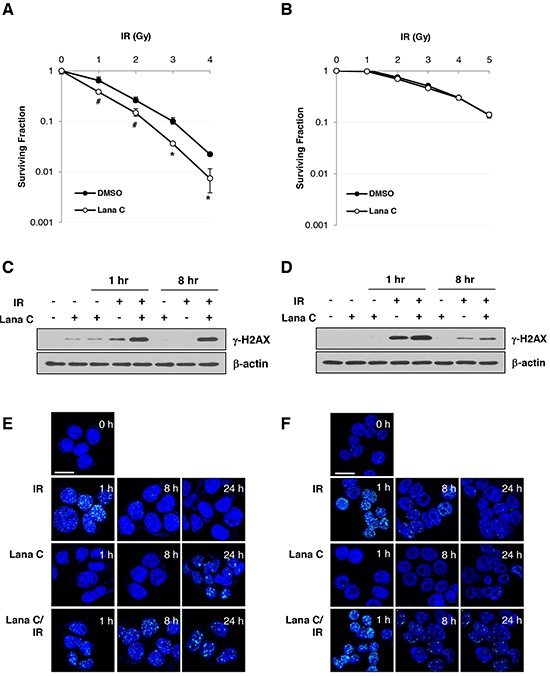
Lanatoside C sensitized HCT116 cell to radiation via suppression of DNA damage repair HCT116 cells **A.** and HT-29 cells **B.** were pretreated with DMSO or 0.2 μM lanatoside C for 16 h and then γ-irradiated. Ten days after irradiation, surviving colonies were counted after staining with crystal violet. *p < 0.01; *p < 0.05. After pretreatment with DMSO or 0.5 μM lanatoside C for 16 h, HCT116 cells **C.** and HT-29 cells **D.** were exposed to 5 Gy γ-irradiation (IR). After additional incubation for 1 or 8 hr, cell were harvested and western blot analysis was performed with an antibody against γ-H2AX. After 1 h pretreatment with 1 μM lanatoside C, HCT116 **E.** and HT-29 **F.** cells were exposed 5 Gy γ-irradiation. Cells were fixed with 4% paraformaldehyde and stained with γ-H2AX antibody. Scale bar: 20 μm.

During the screening of DNA damage response modulator, lanatoside C has been identified as an inhibitor of doxorubicin-induced p53-dependent transcription activation (unpublished result). So, we investigated whether lanatoside C enhanced radiosensitivity of colorectal cancer cells. HCT116 and HT-29 cells were exposed to 0.2 μM lanatoside C for 16 h, followed by γ-irradiation. Eight hours after irradiation, lanatoside C was removed and cells were incubated for an additional 10-14 days and assessed for colony formation. Radiation dose-response curves for lanatoside C plus radiation, which were normalized to account for the cytotoxicity of lanatoside C alone, are shown in Figure 5A and 5B. When combined with radiation, lanatoside C strongly increased radiosensitivity of HCT116, but not of HT-29 cells.

Next, we investigated whether lanatoside C affected the generation and/or elimination of radiation-induced DNA double-strand breaks (DSBs) by measuring γ-H2AX protein level and foci formation. Combination of lanatoside C and radiation generated more DNA damage than single treatments (Figure 5C & 5D, lane 5), which appeared to have a more pronounced effect in HCT116 cells than in HT-29 cells. After 8 h, radiation-induced increase in γ-H2AX level were sustained in the irradiated HCT116 cells pretreated with lanatoside C (Figure 5C, lane 8), suggesting that lanatoside C suppressed DNA damage repair. However, lanatoside C did not inhibit DNA damage repair in HT-29 cells (Figure 5D). As shown in Figure 5E, irradiation-induced γ-H2AX foci were also maintained in lanatoside C-treated HCT116 cells after 8 and 24 h. These results indicated that the radiosensitizing effect of lanatoside C in HCT116 cells was mediated by amplification of DNA damage and suppression of DNA damage repair.

### Lanatoside C inhibited recruitment of 53BP1 to the DNA damage sites through suppression of RNF168 activity

To reveal the mechanism of lanatoside C regulating DNA damage response, we first checked DNA damage response signaling by western blotting. As shown in Supplementary Figure S4A, radiation-induced phosphorylation of H2AX was dramatically increased depending on the concentration of lanatoside C, but the phosphorylation of other signal molecules such ATM and Chk2 was not significantly increased. And it is worth noting that 0.5 μM lanatoside C in combination with radiation depleted p53, p21 and Chk1 proteins (lane 6).

**Figure 6:**
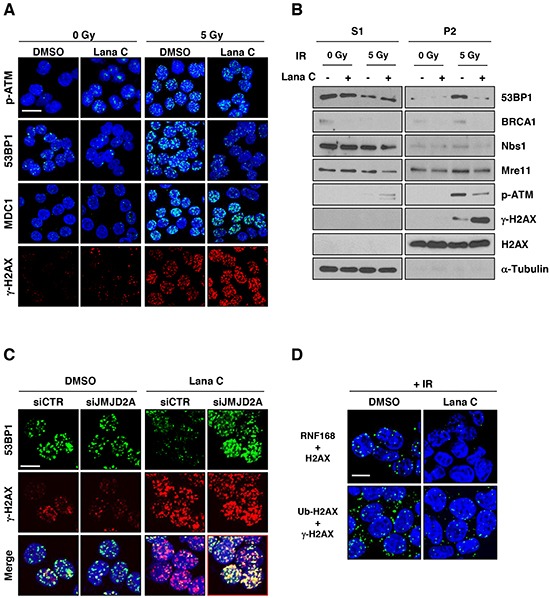
Lanatoside C suppressed 53BP1 recruitment to the DNA damage sites After pretreatment with the indicated concentration of lanatoside C for 16 h, HCT116 cells were exposed to 5 Gy γ-irradiation and incubated for 1 h. **A.** The cells were pre-extracted at 4°C with 0.2% NP-40, followed by fixation with paraformaldehyde. Recruitment of DNA damage-related proteins to DNA damage sites were performed by immunofluorescence with antibodies against γ-H2AX, p-ATM, 53BP1, and MDC1. **B.** The cells were fractionated into soluble (S1) and chromatin-enriched (P2) fractions and analyzed by western blotting for DNA damage-related proteins. **C.** After suppression of JMJD2A expression with siRNA, HCT116 cells were pretreated with DMSO or 0.5 μM lanatoside C, followed by γ-irradiation with 5 Gy. After 1 h incubation, 53BP1 and γ-H2AX foci were observed by immunofluorescence microscopy. **D.** Interaction between RNF168 and H2AX and ubiquitination of γ-H2AX were confirmed with *in situ* proximity ligation assays. Scale bar: 10 μm.

As many proteins regulating DNA damage response are known to form foci at the damaged DNA, we next performed immunofluorescence staining with antibodies against 53BP1, phospho-ATM (S1981), and mediator of DNA damage checkpoint protein 1 (MDC1). Foci formation of γ-H2AX after irradiation was further increased in the cells pretreated with 0.5 μM lanatoside C. However, IR-mediated foci formation of 53BP1 were almost completely blocked by lanatoside C combination. Although the number of phospho-ATM foci was also slightly decreased, MDC1 foci formation was not affected by lanatoside C treatment (Figure 6A). To confirm this result, HCT116 cells were biochemically fractionated and protein retention on damaged DNA sites was investigated. As shown in Figure 6B, 53BP1 was recruited chromatin fraction (P2) after irradiation, which was blocked by lanatoside C pretreatment. Phospho-ATM level in chromatin fraction was also decrease but other molecules did not affected by lanatoside C treatment. As total phospho-ATM was slightly increased in whole cell extract (Supplementary Figure S4B), binding of phospho-ATM to chromatin could be affected by lanatoside C.

Recent studies show that formation of 53BP1 foci is dependent on RNF8- and RNF168-mediated JMJD2A ubiquitination and degradation at the damaged DNA sites [18]. To determine whether lanatoside C-dependent suppression of 53BP1 foci formation was related to JMJD2A, we examined 53BP1 foci formation in JMJD2A-knockdown cells after irradiation. As shown in Figure 6C, JMJD2A suppression restored 53BP1 foci formation which was inhibited by lanatoside C (yellow foci). Colocalization of 53BP1 and γ-H2AX foci was confirmed by analysis of fluorescence peak distance from images (Supplementary Figure S4C, right panel). RNF8 and RNF168 also ubiquitinated histone H2A at Lys13-15 and Lys119 following generation of DSBs, which is followed by recruitment of BRCA1 or 53BP1 [19, 20]. We investigated the interaction between RNF168 and H2AX and confirmed ubiquitination of γ-H2AX using *in situ* proximity ligation assays (PLAs). Lanatoside C eliminated the irradiation-induced interaction between RNF168 and H2AX and reduced ubiquitination of H2AX (Figure 6D and Supplementary Figure S5). These results suggested that lanatoside C-mediated inhibition of RNF8 or RNF168 may contribute to prevention of 53BP1 foci formation by reducing JMJD2A ubiquitination/degradation and histone ubiquitination in the damaged DNA region.

### Lanatoside C enhanced radiation-induced tumor growth delay *in vivo*

**Figure 7:**
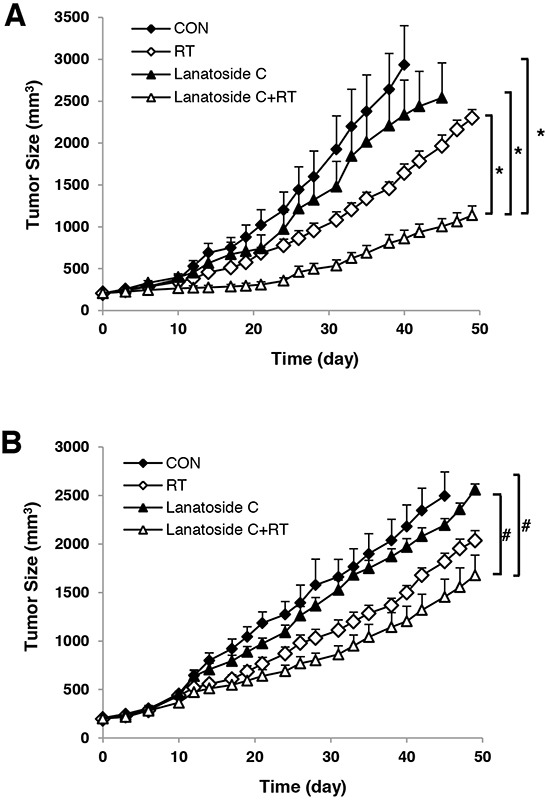
Lanatoside C improved radiation-induced tumor growth delay *in vivo* HCT116 **A.** and HT-29 **B.** cells (1×10^6^ cells) were implanted subcutaneously in nude mice. Tumors were randomized at 200 mm^3^ into four groups and treated with vehicle control (CON), lanatoside C alone (6 mg/kg by intraperitoneal injection), radiotherapy (RT, 8 Gy), or combination treatment with lanatoside C and radiotherapy. Tumors were measured three times per week and followed until they reached 3,000 mm^3^ or 49 days. *p < 0.01; ^#^*p < 0.05*

To confirm the radiosensitizing effects of lanatoside C *in vivo*, we generated mouse xenograft models via inoculation with HCT116 or HT-29 cells. Tumor growth was measured after administration of γ-irradiation with or without lanatoside C. In the xenograft model generated with HCT116 cells, irradiation alone inhibited tumor growth by 44.92 ± 5.15% and irradiation plus lanatoside C inhibited growth by 77.76 ± 3.03% on day 40 (Figure 7A). In the HT-29 xenograft model, tumor growth was inhibited by 21.45 ± 6.64% with irradiation alone and 41.31 ± 6.84% with lanatoside C plus irradiation on day 45 (Figure 7B). Lanatoside C alone did not significantly inhibit tumor growth; however, lanatoside C improved the efficacy of radiotherapy *in vivo*, particularly in the HCT116 xenograft.

## DISCUSSION

The current study demonstrated that treatment with lanatoside C led to dose-dependent cytostatic or cytotoxic responses in two colorectal cancer cell lines, HCT116 and HT-29. However, lanatoside C strongly enhanced the radiosensitivity of HCT116 but not HT-29 cells *in vitro*. As cells are most radiosensitive in the M and G2 phases [21], cell cycle arrest in the G2/M phase by lanatoside C in HCT-116 cell could be responsible for the difference in radiosensitization (Supplementary Figure S1). Not only this, lanatoside C increased cell sensitivity to radiation by inhibiting DNA damage repair (Figure 5C) in HCT-116 cell, as has been reported for other cardiac glycosides including bufalin, ouabain, and huachansu [22–24].

Induction of DNA DSBs triggers a cascade of protein modification and relocalization. Phosphorylation of H2AX causes recruitment of downstream factors, such as the E3 ubiquitin ligases RNF8 and RNF168, leading to the formation of K63-linked polyubiquitin chains on histones at DSBs. This ubiquitination cascade is responsible for the localization of repair mediators, such as 53BP1 and BRCA1, to sites of DNA damage [19, 20, 25–27]. Localization of 53BP1 to DSBs involves recognition of H2A ubiquitination at Lys15 and requires dimethylation of histone H4 on Lys20 (H4K20me2) via the 53BP1 tandem Tudor domain [28, 29]. In this study, lanatoside C suppressed the formation of radiation-induced 53BP1 foci (Figure 6A) and retention at the sites of DNA damage (Figure 6B). Mallette *et al.* [19] shows that the Tudor domain containing lysine demethylases JMJD2A and JMJD2B, which bind to H4K20me2, becomes polyubiquitinated and degraded in response to DNA damage and that this process requires E3 ligase activity of both RNF8 and RNF168. We demonstrated that knockdown of JMJD2A significantly increased the formation of 53BP1 foci in lanatoside C-treated cells (Figure 6C) and lanatoside C dramatically reduced RNF168-mediated H2AX ubiquitination (Figure 6D). Thus, we suggested that the lanatoside C-induced radiosensitizing effect is mediated by suppression of the RNF8/RNF168-mediated JMJD2A or H2AX ubiquitination, which required for 53BP1 recruitment and DNA damage repair.

Autophagy is characterized by the presence of cytoplasmic vesicles and is involved in a variety of cellular functions, including development, nutrient sensing responses, and cell death [30]. Importantly, autophagy has become a vital mechanism for anti-cancer treatments [31, 32]. A recent study shows that many cardiac glycosides induce autophagy in human non-small lung cancer cells through activation of the AMP-activated protein kinase (AMPK) pathway and Erk1/2 activation, which leads to autophagic cell death [33]. In our study, lanatoside C also induced autophagy through activation of Erk1/2 and JNK1/2 signaling (Figure 2 and 3). However, suppression of autophagy by depletion of Atg5 and Beclin 1 (key regulators of autophagy) could not reduce lanatoside C-mediated growth inhibition (Supplementary Figure S2). Loss of mitochondrial membrane potential (MtMP) is another major determinant of cell death, and we found a significant decrease in MtMP in lanatoside C-treated colorectal cancer cells (Figure 4). Badr *et al.* also shows that lanatoside C causes loss of MtMP and decreases intracellular ATP levels as early as 2 h after treatment in glioblastoma cell lines. They suggest that lanatoside C-induced autophagy is triggered by a decrease in MtMP followed by early depletion of ATP [34]. Similarly, lanatoside C resulted in loss of MtMP and induction of autophagy in colorectal cancer cell lines, and disruption of K^+^ homeostasis by inhibition of Na^+^/K^+^-ATPase activity was a key factor in the induction of depolarization of mitochondria. As damaged mitochondria becomes autophagosomes or mitopototic bodies, which are degraded through mitoptosis [16], we speculated that autophagic process induced by lanatoside C might be induced to eliminate the damaged mitochondria.

## MATERIALS AND METHODS

### Cells and reagents

Human colorectal adenocarcinoma cancer cell lines HCT116 and HT-29 were obtained from the Korean Cell Line Bank (KCLB, Seoul, Korea). All cells were maintained in RPMI-1640 medium (WELGENE Inc., Daegu, Korea) supplemented with 10% heat-inactivated fetal bovine serum (FBS) (Omega Scientific, Inc., Tarzana, CA, USA) and 100 U/ml penicillin/streptomycin. Lanatoside C and bafilomycin A1 was obtained from Santa Cruz Biotechnology (Santa Cruz, CA, USA). U-0126 and SP600125 were purchased from Assay Designs (Ann Arbor, MI, USA). MitoTracker^R^ Red CMXROS, 5,5′,6,6′-tetrachloro-1,1′,3,3′-tetraethylbenzimidazolylcarbocyanineiodide (JC-1) and 4-Di-1-ASP (4-(4-(Dimethylamino)styryl)-N-Methylpyridinum Iodine (DASPMI) were purchased from Life Technologies (Grand Island, NY, USA).

### WST cell viability assay

HCT116 and HT-29 cells were exposed to various concentrations of lanatoside C for 48 h, and cell viability was evaluated using the Ez-Cytox Cell Viability, Proliferation & Cytotoxicity Assay kit (Daeil Lab Service, Seoul, Korea) according to the manufacturer's instructions.

### Cell cycle analysis

Cells were treated with the indicated amount of lanatoside C for 24 h, harvested by trypsinization and fixed with 70% ethanol at −20°C for overnight. Fixed cells were stained with propidium iodide (50 μg/ml) containing 50 μg/ml of RNase A (Sigma), and analyzed by flow cytometry for cell cycle profile using a FACSCalibur™ system (BD Biosciences, San Jose, CA, USA) and data were analyzed using CellQuest™ Pro software (BD Biosciences).

### Autophagy assays

For quantitative analysis of fluorescenct autophagosome, GFP-LC3-expressing HCT-116 cell was established by infecting retrovirus made with pBABEpuro GFP-LC3 (a gift from Jayanta Debnath, Addgene plasmid # 22405). After 48 h incubation with 0.5 μM lanatoside C, the percentage of GFP-LC3 positive cells with GFP-puncta was assessed by counting a minimum of 100 cells with fluorescence microscopy (Axio Observer D1; Carl Zeiss MicroImaging, Jena, Germany).

### Annexin V staining

After 24 h treatment with lanatoside C, cells were harvested by trypsinization and apoptotic cells were detected using Annexin V-FITC Apoptosis Detection Kit (Biovision, Milpitas, CA, USA) by flow cytometry.

### Measurement of mitochondrial membrane potential

HCT116 cells and HT-29 cells were treated with lanatoside C for 3 h, followed by staining with 10 μM DASPMI for 30 min. Cells were harvested by trypsinization and suspension in phosphate-buffered saline (PBS) containing 10 μM D-Glucose. Mitochondrial membrane potential was analyzed by flow cytometry. For fluorescence image analysis, cell were stained with 20 nM MitoTracker Red CMXROS or 10 mg/ml JC-1 for 30 min and changes in mitochondrial morphologies and mitochondrial membrane potential were observed.

### Measurement of oxygen consumption rate

Rates of oxygen consumption (OCR) were measured using an Extracellular Flux Analyzer (XF-24, Seahorse Biosciences, North Billerica, MA, USA) according to the manufacturer's instructions. Briefly, cells were seeded into 24-well plates (4×10^4^ cells/well) and cultured overnight in RPMI media with 10% FBS. Cells were washed with media and treated with the indicated dose of lanatoside C for 3 h. Cells were washed with Seahorse assay media (1 μM pyruvate, 25 μM glucose) and incubated in the same media for 1 h at 37°C without CO_2_. Cells were loaded onto the XF-24 analyzer to measure OCR in short and repeated intervals. Baseline cellular OCR was measured, followed by ATP-linked respiration in the presence of oligomycin (1 μg/ml). Next, carbonyl cyanide-p-trifluoromethoxyphenyl-hydrazon (FCCP) (1 μM) was added to measure maximal respiratory capacity. Lastly, antimycin A (2 μM), an inhibitor of complex III and I, was added to block function of the electron transport chain. Mixing, waiting, and measuring times were 3, 2, and 3 min, respectively.

### Irradiation

γ-radiation was delivered using a dual-source ^137^Cs unit at a dose rate of 3.2 Gy/min with a GC-3000 Elan irradiator (MDS Nordion, Canada).

### Clonogenic cell survival assay

Cells were seeded into 60-μm culture plates (2×10^2^ – 3×10^3^ cells/plate) and allowed to attach for 24 h prior to treatment. Cells were treated with DMSO or 0.2 μM lanatoside C for 16 h, followed by γ-irradiation. After 10-14 days of incubation, colonies were fixed with ice-cold methanol and stained with 0.4% crystal violet in 20% ethanol. The colonies with more than 50 cells were counted. The survival fraction was calculated as followed: Survival fraction = colonies counted / (cell seeded × plating efficiency / 100). Cell survival was normalized against cytotoxicity induced by lanatoside C and results were from at least three independent experiments.

### In situ extraction and immunofluorescence

Cells were cultured on 18×18-mm cover glasses, and cell extraction was performed with a slight modification of a previous report [35]. Cells were extracted *in situ* by incubating the cover glasses in fractionation buffer [50 μM HEPES (pH7.5), 150 μM NaCl, 1 μM EDTA, 0.2% Nonidet P-40] for 20 min on ice. The buffer was removed, and the procedure was repeated for two additional incubations on ice for 10 and 5 min, respectively. Cells were fixed in 4% paraformaldehyde for 10 min and permeabilized with 0.5% Triton X-100 for 10 min. Intact cells were fixed without extraction and blocked in PBS containing 10% FBS for 30 min. Cells were incubated with antibodies against γ-H2AX (Ser139), phospho-ATM (Ser1981), MDC1 (Millipore, Billerica, MA, USA), and 53BP1 (Santa Cruz Biotechnology) overnight at 4°C and then with fluorescein isothiocyanate (FITC)-conjugated secondary antibodies (Invitrogen) for 1 h at room temperature. After counterstaining with 4′, 6-diamidino-2-phenylindole (DAPI), immunofluorescence images were captured with a laser-scanning confocal microscope (LSM710; Carl Zeiss MicroImaging).

### Western blot analysis

Western blot was performed following a standard protocol. Antibodies were obtained as follow; p53, p21, Chk1, p62, PARP-1, β-actin (Santa Cruz Biotechnology), phospho-p53 (Ser15), phospho-Chk2 (Thr68), phospho-Erk1/2, Erk1/2, phospho-JNK, JNK1/2, phospho-p38, p38, Caspase 3, H2AX (Cell Signaling Technology), phospho-ATM (Ser1981), Chk2, γ-H2AX (Ser139) (Millipore), ATM (Epitomics, Burlingame, CA, USA), and LC3 (Novus Biologicals, Littleton, CO, USA). Chemiluminescence was detected using enhanced chemiluminescence detection reagents.

### Biochemical fractionation

Biochemical fractionation was performed with slight modifications [35]. Briefly, the cells were washed twice with ice-cold PBS and harvested. Cell pellets were resuspended for 15 min on ice in 200 μl extraction buffer [50 μM HEPES (pH7.5), 150 μM NaCl, 1 μM EDTA] containing 0.1% Triton X-100 and supplemented with protease inhibitor cocktail and phosphatase inhibitors. Following centrifugation at 13,000 rpm for 3 min, the supernatant was collected (S1), and pellets were further extracted for 15 min on ice using the same buffer and collected. The pellets were further incubated in 200 μl of extraction buffer without Triton X-100 supplemented with 200 μg/ml RNase A for 30 min at 25°C. Following centrifugation at 13,000 rpm for 3 min, the supernatant was separated from the pellet. The pellet was resuspended in PBS buffer supplemented with 1% SDS, boiled for 10 min, and sonicated for 10 sec (Q500, Qsonica, LLC, Newtown, CT, USA) (P2). S1 and P2 fractions were used for western blot analysis with antibodies against 53BP1, BRCA1, NBS1, MRE11, α-tubulin (Santa Cruz Biotechnology), phospho-ATM (Ser1981), and γ-H2AX.

### Small interfering RNAs (siRNAs)

The siRNA duplexes with the following sequences were synthesized by Genolution, Inc. (Seoul, Korea): Erk1 (5′-UUAGAGAGCAUCUCAGCCAGAAUGC-3′), Erk2 (5′-AAGAGGAUUGAAGUAGAACAGdTdT-3′), JNK1 (5′-GAAGCUCCACCACCAAAGAUCUU-3′), JNK2 (5′-GACUCAACCUUCACUGUCCUAUU-3′), ATG5 (5′-GGAAUAUCCUGCAGAAG AAUU-3′), Beclin 1 (5′-GCUCAGUAUCAGAGAGAAUUU-3′), and JMJD2A (5′-GUAUGAUCUU CCAGACUUAUU-3′). Cells in 60-mm culture plates were transfected with 20 nM siRNA using Lipofectamine RNAiMAX reagent (Invitrogen) according to the manufacturer's instructions.

### In situ proximity ligation assay (PLA)

*In situ* PLA was performed according to the manufacturer's instructions (Olink Bioscience, Uppsala, Sweden). Briefly, cells cultured on 18×18-mm cover glasses were fixed in 4% paraformaldehyde for 15 min, permeabilized with 0.5% Triton X-100 for 10 min, and blocked at 37°C for 30 min. Co-incubations with anti-RNF168 (Bioss, Woburn, MA, USA) and anti-H2AX or with anti-Ub-H2AX (K119) (Cell Signaling Technology) and anti-γ-H2AX (Millipore) were performed overnight at 4°C. After removal of unbound primary antibodies, cells were incubated with proximity probes (anti-rabbit PLUS and anti-mouse MINUS) (Olink Bioscience) for 1 h at 37°C. Ligation was performed for 30 min at 37°C, followed by polymerization for 2 h at 37°C. The cells were counterstained with DAPI, and immunofluorescence images were captured with a laser-scanning confocal microscope.

### Colorectal cancer xenograft studies

Male 4-week-old Balb/c nude mice were used for this study. Animals were maintained at animal care facilities, and food and water were supplied *ad libitum*. Studies were conducted under guidelines for the use and care of laboratory animals and approved by the Institutional Animal Care and Use Committee of the Korea Institute Radiological and Medical Sciences. Tumors were generated by subcutaneous injection of 1×10^6^ cells into the right hind leg of Balb/c nude mice. When tumors reached a mean diameter of 7.5-8 mm, mice (*n* = 6 per group) were randomly assigned to one of the four experimental groups. Radiation treatment was administered when the tumors had grown to a mean diameter of 7.5-8 mm (which represented Day 0). The tumor-bearing legs were treated with a single dose of 8 Gy using Theratron 780 (AECL, Ontario, ON, Canada). When tumors were a mean diameter of 7.5-8 mm, PBS or 6 mg/kg of lanatoside C were injected intraperitoneally one time per day for five days. In the radiation plus lanatoside C group, lanatoside C was administered 6 h following radiation (Day 0). Tumor growth was assessed by regular measurement of orthogonal tumor diameter until the diameter reached at least 20 mm. The length and width of tumor sizes were determined two or three times per week with calipers. Tumor volumes (mm^3^) were calculated according to the following formula: length×width^2^×0.523.

### Statistical analysis

All results were analyzed for statistical significance that was assessed using a Student's *t*-test. A *p* value of less than 0.05 compare with the control was considered statistically significant.

## SUPPLEMENTARY FIGURES LEGENDS


