# Supplementary material for: Lanatoside C suppressed colorectal cancer cell growth by inducing mitochondrial dysfunction and increased radiation sensitivity by impairing DNA damage repair
Source: Oncotarget. 2016 Jan 7;7(5):6074–87. doi: 10.18632/oncotarget.6832 (PMC4868741; doi:10.18632/oncotarget.6832)
Supplement: Supplementary file 1 [file oncotarget-07-6074-s001.pdf]

SUPPLEMENTARY FIGURES LEGENDS

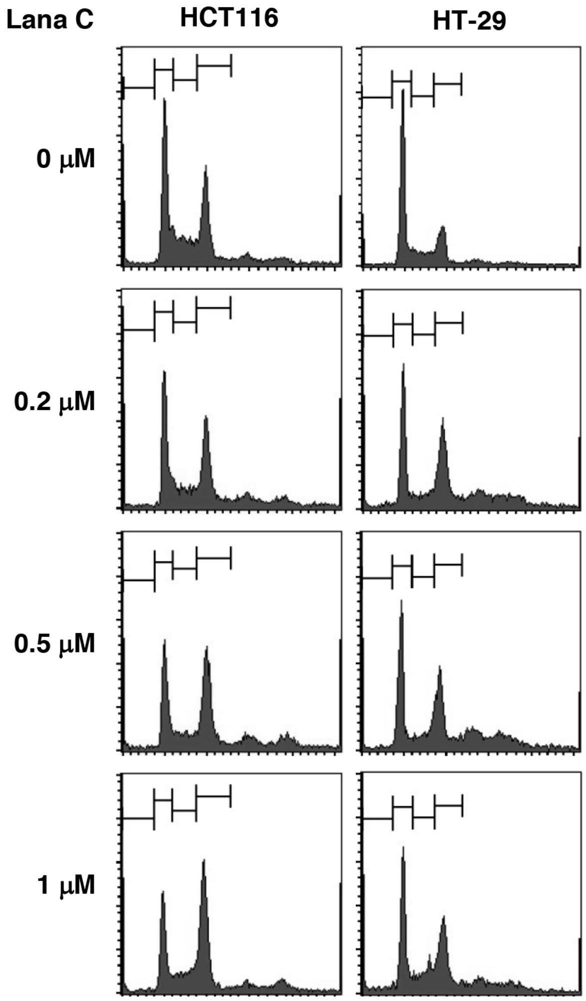

Supplementary Figure S1: Lanatoside C induced G2/M arrest in a dose-dependent manner. HCT116 and HT-29 cells were treated with the indicated concentration of lanatoside C for 24 h, followed by flow cytometric analysis.

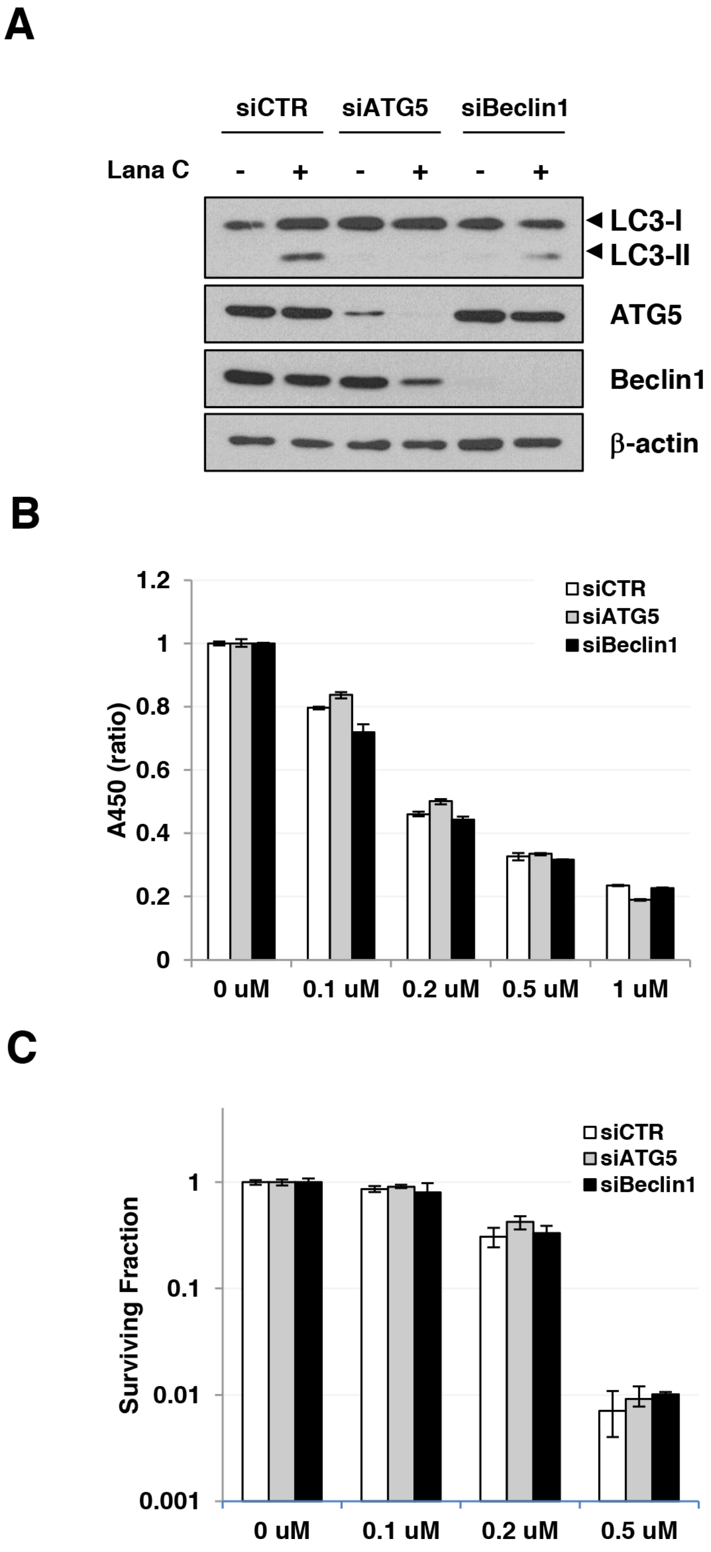

Supplementary Figure S2: Anti-cancer effect of lanatoside C was not mediated by autophagy. After transfection with control siRNA (siCTR) or the indicated siRNAs, **A.** HCT116 cells were treated with 1 μM lanatoside C for 24 h. Expression of LC3, ATG5, and Beclin1 were analyzed by western blot. **B.** HCT116 cells were treated with the indicated concentration of lanatoside C for 48 h, viable cell was quantified. **C.** HCT116 cells were treated with the indicated concentration of lanatoside C for 24 h. Cell death was analyzed by clonogenic survival assay after 10 days of treatment. Surviving colonies were counted after staining with crystal violet.

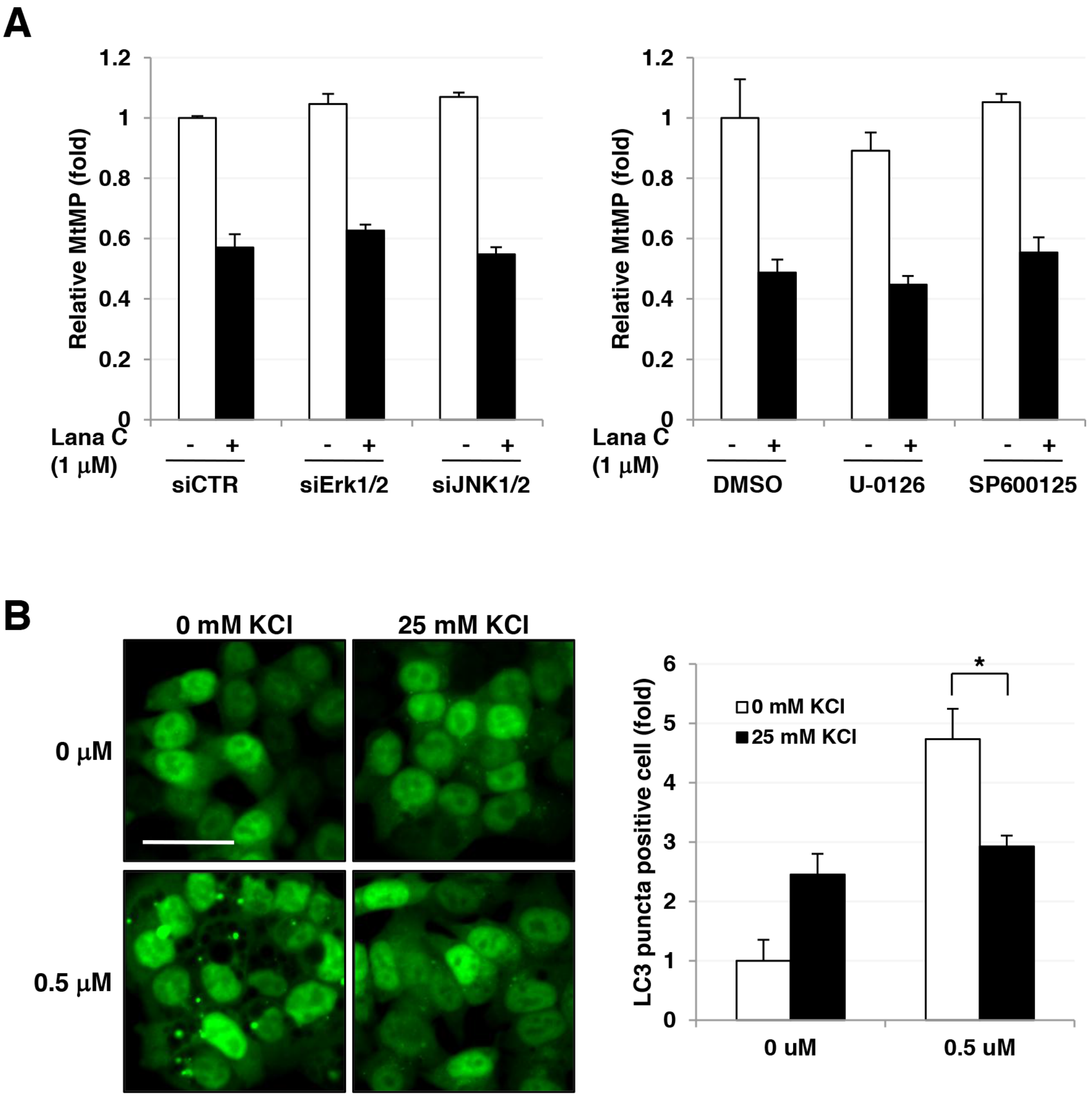

Supplementary Figure S3: Lanatoside C-induced disruption of K^+ homeostasis led to autophagy. A. After transfection with siRNAs (left panel), or pretreatment with Erk1/2 inhibitor (10 µM U-0126) or JNK1/2 inhibitor (10 µM SP600125) for 1 h (right panel), the cells were treated with 1 µM lanatoside C. After 3 h, cells were incubated with 10 µM DASPMI for 30 min and mitochondrial membrane potential was measured with flow cytometry. **B.** After pretreatment with 25 mM KCl, cells were treated with 0.5 µM lanatoside C for 48 h. LC3 puncta formation was observed by fluorescence microscopy. Representative images were obtained at 200× magnification. Scale bar: 20 µm. *, $p < 0.05$.

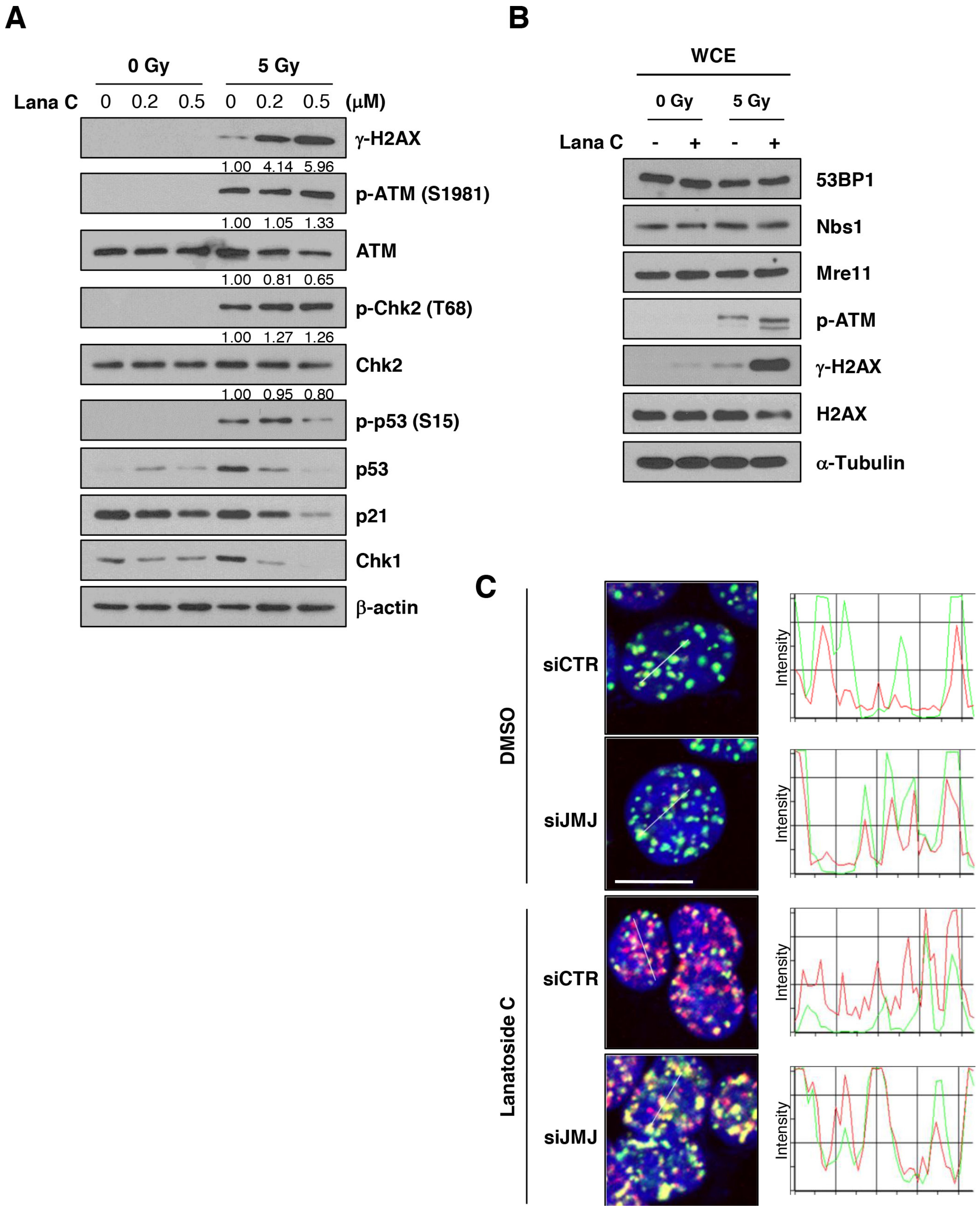

Supplementary Figure S4: Lanatoside C-mediated inhibition of 53BP1 recruitment was mediated by JMJD2A retention at DNA damage sites. A. HCT116 cells were treated with 0, 0.2 or 0.5 µM lanatoside C for 16 h, followed by γ-irradiation (5 Gy). After 1 h incubation, the DNA damage signaling was examined by western blot analysis. Densitometric analysis of protein bands was performed with Image J program. **B.** Total protein level was analyzed from cells used for fractionation experiment in Figure 6B. Whole cell extracts were prepared with 1 X PBS containing 1% SDS and analyzed by western blotting for DNA damage-related proteins. **C.** Using representative cell images in figure 6C, fluorescent intensity of γ-H2AX (red) and 53BP1 (green) along the line in the image were analyzed with Image-Pro Plus and displayed as a graph (right panel). Peaks in same location represent colocalization of two proteins. Green line: 53BP1, red line: γ-H2AX. Scale bar: 10 µm.

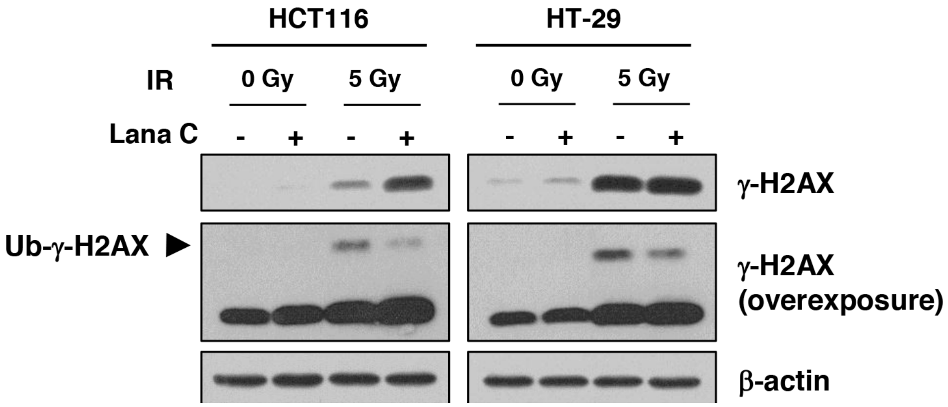

Supplementary Figure S5: Lanatoside C suppressed ubiquitination of radiation-induced γ-H2AX protein. After pretreatment with DMSO or 0.5 μM lanatoside C for 16 h, HCT116 and HT-29 cells were exposed to 5 Gy γ-irradiation and further incubated for 1 h. Western blot analysis was performed with antibodies against γ-H2AX and then overexposed in order to detect higher molecular weight form of ubiquitinated γ-H2AX (arrow).
